# Demography and Histologic Pattern of Laryngeal Squamous Cell Carcinoma in Kenya

**DOI:** 10.1155/2014/507189

**Published:** 2014-01-21

**Authors:** Owen Pyeko Menach, Asmeeta Patel, Herbert Ouma Oburra

**Affiliations:** ^1^Department of Surgery, ENT Division, University of Nairobi, P.O. Box 330-00202, Nairobi, Kenya; ^2^ENT Head & Neck Department, Kenyatta National Hospital, P.O. Box 20723-00202, Nairobi, Kenya

## Abstract

*Background*. Laryngeal squamous cell carcinoma is a common head and neck cancer worldwide. *Objective*. To determine the demographic characteristics of patients with laryngeal cancer, establish their tumor characteristics and relate it to their smoking and alcohol ingestion habits. *Methods*. Fifty cases and fifty controls were recruited of matching age, sex, and region of residence. History and pattern of cigarette smoking and alcohol ingestion was taken and analyzed. *Results*. 33 (66%) of the cases and 3 (6%) among controls were current cigarette smokers. 74% had smoked for more than 30 years, *P* < 0.0001 OR 21.3 (95% CI: 2.6–176.1). There was a male predominance (96%) and most cases (62%) were from the ethnic communities in the highland areas of Kenya predominantly in Central and Eastern provinces. Very heavy drinkers had increased risk of *P* < 0.0001 OR, 6.0 (95% CI: 1.957–18.398) and those who smoked cigarettes and drank alcohol had poorly differentiated tumors G3, *P* < 0.001, OR 11.652 (95% CI 2.305–58.895), and G4, *P*=0.52 OR 7.286 (95% CI 0.726–73.075). They also presented with advanced disease (73.6%). *Conclusion*. Cigarette smoking and alcohol ingestion are strong risk factors for development of late stage and poorly differentiated laryngeal squamous cell carcinoma in Kenya.

## 1. Background

The commonest causes of death in Kenya are infectious diseases followed closely by cardiovascular illnesses and cancer in that order [1]. Cancer cases in Kenya have however been steadily rising due to the increasing prevalence of cigarette smoking which is a known cause of various neoplasias, more so the upper aerodigestive tract and lung tumors [1, 2]. This rise has been documented and published by the Nairobi Cancer Registry [3], but it is not thought to depict the accurate situation on the ground because cancer diagnosis and notification from health institutions are not as meticulous as desirable. This increase has not been captured in local studies especially with regard to head and neck cancer in general and laryngeal carcinoma in particular.

From previous published work, cigarette smoking and alcohol ingestion have been shown to be major risk factors for laryngeal squamous cell carcinoma in this locality as seen in other populations [4]. The incidence of this cancer may increase considering the rising prevalence of smoking in Kenya especially among men in the 45–49 years of age bracket [1, 2]. Moreover, it is also quite worrying that 13% of schooling children smoke cigarettes and, just like in adults, males smoke more than females [5, 6]. If not checked, there is likelihood of increased cancer burden corresponding to the shifting trends and rising prevalence of cigarette smoking in this population.

Globally, about a fifth of the world's population smoke cigarettes and these figures are increasing exponentially due to extensive and aggressive marketing done by cigarette manufacturing companies [7]. The smoking pattern in the world seems to vary in the various continents with Russia and China leading to the prevalence of the disease. This trend seems to be reducing in the West while it is increasing in the developing world though the prevalence is still higher in the Western countries, Europe, and Asia [7, 8].

Concurrent cigarette smoking and ingestion of ethanol have been shown to increase mucosal penetration of tobacco carcinogens as well as production of carcinogenic metabolites in the upper aerodigestive tract [9, 10]. The risk imparted by both factors has been shown to be synergistic thus increasing the risk of squamous cell carcinoma by many fold [4, 9, 10]. However, alcohol as an independent risk factor for laryngeal cancer remains controversial [10]. The relation between histologic differentiation of squamous cell carcinoma with cigarette smoking and alcohol ingestion has been documented [11]. These risk factors have been shown to predispose to poorly differentiated forms and therefore have a poorer outcome than more differentiated ones though other authors dispute this finding [11, 12]. Overall, the prevalence of cigarette smoking and alcohol ingestion is increasing, but its relation to cancer has not been clearly elucidated with regard to laryngeal squamous cell carcinoma in this population.

## 2. Objective 

The objective of this study was to determine the demographic characteristics of patients with laryngeal squamous cell carcinoma, establish their tumor characteristics, and relate it to their cigarette smoking and alcohol ingestion habits.

## 3. Study Design

This was a hospital based case-control study.

## 4. Sample Size Calculation

The sample size was calculated using the Hennekens and Buring [13] formula for comparing two proportions.

## 5. Methodology

The study was conducted between March and May, 2011, at the Kenyatta National Hospital.

### 5.1. Recruitment of Study Patients

A total of 50 cases whose histology had been confirmed to be squamous cell carcinoma were recruited for the study. 78% were recruited from the Otorhinolaryngology Head and Neck Department whereas 22% were recruited from the Radiation-Oncology Department. All the cases were staged clinically, endoscopically, and radiologically by computerized tomography scanning and finally discussed in the Otorhinolaryngology Head and Neck Tumor Board. Six cases that had nonsquamous cell histologies were excluded from the study whereas two others were declined.

### 5.2. Recruitment of Controls

A total of 50 controls were recruited. 72% were recruited from the Orthopedic Wards whereas 28% were recruited from the Orthopedic Clinic. Among the controls, three patients were found to have dysphonia whereas one had a neck swelling and was referred to the otorhinolaryngology clinic for detailed assessment. 62% had been admitted with fractures and dislocations, 25% with benign spinal ailments, and the rest had inflammatory and infectious ailments. Controls were matched by age with the cases within a range of five years.

### 5.3. Establishment of Demographic Data, Presence and Nature of Smoking Habit and Alcohol Ingestion

The patient's demographic data including age, sex, and region of origin, occupation as well as smoking and alcohol intake habits were documented. Symptomatology and nasolaryngoscopy data were obtained from the control subjects with a focus to rule out laryngeal cancer. The controls that did not have symptoms and nasolaryngoscopy evidence of laryngeal cancer were included in the study. Their alcohol intake habits, including the type, were categorized as per NIAAA [14] guidelines while cigarette smoking habits were classified in pack-years in both groups.

## 6. Data Management and Analysis

All the information was recorded in a data collection questionnaire. This data was then entered into a password protected Microsoft Access database. Quality control was performed by comparing the data entered into the Microsoft Access database with the hard copy forms, identifying inconsistent ones and making appropriate corrections. The data was thereafter exported to SPSS 17.0 statistical package which was used for subsequent data analysis. Results were presented in tables, graphs, and pie charts. Mean, median, and standard deviation were computed and used to summarize continuous variables while simple counts, frequencies, and proportions were used to summarize nominal variables. Chi-squared and Fisher's exact tests were used to detect associations between nominal variables whereas ANOVA and *t*-tests were used to detect associations between continuous and nominal variables. Multivariate logistic regression methods were used to detect associations between continuous variables.

## 7. Results

### 7.1. Age Distribution

The youngest age for the cases was 42 years while the oldest was 84 years with a mean age of 63 years. There was no statistically significant difference between the case and control group whose mean age was 61 years (*P* = 0.297).

### 7.2. Sex Distribution

Out of the 50 cases, only 2 were female (4%) whereas the rest were male (96%) suggesting that this is predominantly a male disease.

### 7.3. Region of Origin

**Figure 1:**
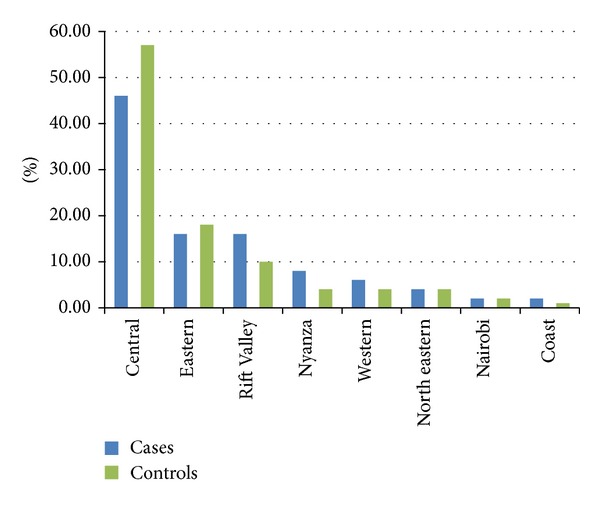
Distribution of cases and controls by region.

The highland areas of Central province (46%) and Eastern province (16%) were the most affected geographical areas. The lowlands of Rift Valley, Nyanza and Western followed suit with 16%, 8%, and 6%, respectively (Figure 1). The least number of cases was seen in Nairobi province (*P* = 0.281). Some cases from Nyanza and coast province were controlled against controls recruited from other regions due to unavailability of the suitable age and sex. This study did not determine to what extent ethnicity contributed to regional biases.

### 7.4. Occupation

**Figure 2:**
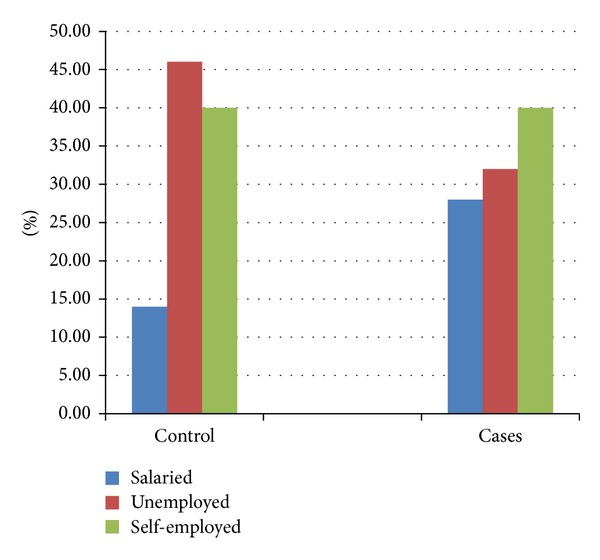
Occupation among cases and controls.

An equal proportion of cases and controls were self-employed whereas more of the controls were unemployed compared to the cases. Twice as much of the cases were salaried compared to the controls although these findings were not statistically significant (*P* = 0.166) (Figure 2).

### 7.5. Education

**Figure 3:**
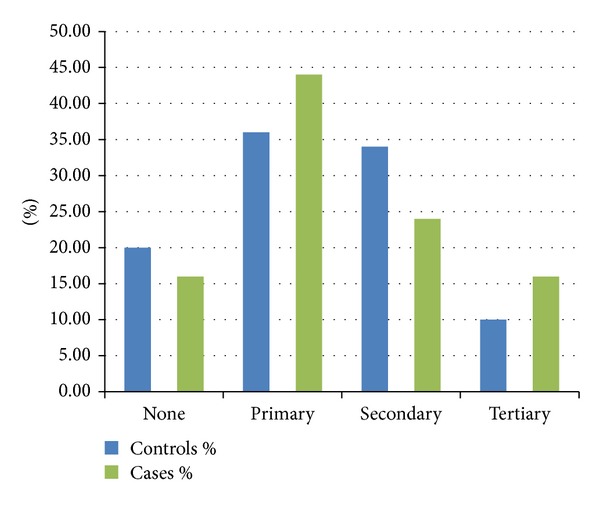
Levels of education among cases and controls.

Majority of the cases had attained primary education compared to controls whereas other levels of education were comparable (*P* = 0.57) (Figure 3).

In general, the characteristics of the cases in terms of sex, age, region of origin, occupation, and level of education were similar with the distribution among controls.

### 7.6. Smoking History

The prevalence of the respondents who lived with someone who smoked in the house was comparable between cases and controls at 3% and 2%, respectively. The *P* values for living with a smoker and smoking in the house were 0.558 and 0.307, respectively (Table 1).

Similarly, the mean age of starting cigarette smoking among the cases was 20.18 whereas it was 25 among the controls (*P* = 0.044). Those who started smoking below the age of 20 years had *P* < 0.0001, OR of 31.733 (95% CI: 8.74–115.04) whereas those who started smoking between 21 and 40 years of age had *P* < 0.001, OR of 7.727 (95% CI: 2.409–24.787). *P* value for those who began smoking after 40 years was 1.00. Those who smoked more than 30 pack-years among the cases had higher risks compared to controls, OR of 21.3 *P* < 0.0001, 95% CI 2.6–176.1). This confirms the fact that longer duration and greater intensity of cigarette smoking portend a great risk for laryngeal SCC.

### 7.7. Alcohol Consumption

38 (76%) cases consumed alcohol compared to 29 (58%) controls. This was statistically significant, *P* = 0.05, OR of 2.3 (95% CI: 1.0–5.4), though it was more pronounced among those who were very heavy drinkers (*P* = 0.002 OR 6.0 (95% CI: 11.957–18.398).

### 7.8. Tumor Characteristics and Stage of Tumor

In this study, majority of the cases had histological diagnosis of well differentiated (G1) squamous cell carcinoma followed by moderately differentiated (G2), and poorly differentiated (G3) and the least was undifferentiated carcinoma (G4).

Majority of the cases presented in stages 3 and 4 of the disease (Table 2).

### 7.9. Risk Factors in relation to Histological Grade

On analyzing the histological grades against the risk exposures being studied, 4 cases smoked but did not drink alcohol and developed G1 carcinoma. Of note is that none of those who smoked without ingesting alcohol developed poorly differentiated cancer and none of those who drank alcohol without smoking cigarettes developed G1 carcinoma. This relation was of statistical significance, *P*  value < 0.0001, OR of 21.333 (95% CI 2.227–204.364), when compared to controls.

### 7.10. Cigarette Smoking and Alcohol Ingestion

There was a statistically significant increased risk for laryngeal squamous cell carcinoma on all histological grades except for G1 tumors among those who smoked cigarettes and consumed alcohol concurrently, but, notably, they were more predisposed to develop poorly differentiated tumors G3 and G4 (Table 3).

Multivariate logistic regression was performed on variables that had significant *P* values. The variables included were being a current smoker (*P* ≤ 0.0001), duration since stopping smoking (*P* = 0.029), age of smoking debut (*P* = 0.044), cumulative pack-years (*P* < 0.0001), duration of smoking (*P* < 0.0001), prevalence of alcohol intake (*P* = 0.05), drinks taken per week (*P* = 0.028), G1 tumors (*P* < 0.001) and G2 tumors (*P* 0.022). Only being a current smoker OR of 14.576 (95% CI 2.624–80.979), and long duration of smoking, OR of 7.312 (95% CI 1.619–33.024), were independently associated with increased risk for laryngeal squamous cell carcinoma in this population.

## 8. Discussion

According to the results of this study, majority of the cases of laryngeal squamous cell carcinoma were elderly males. This is comparable to studies done elsewhere since men tend to consume more alcohol and smoke more cigarettes than females as is found in Kenyan surveys [1, 2, 7]. In these surveys, about 2% of women used tobacco in its various forms whereas 1% smoked cigarettes which translate to the low prevalence of laryngeal squamous cell carcinoma among females. Studies in other populations show male prevalence of laryngeal squamous cell carcinoma as high as 100% [15]. Gallus et al. [16] carried a study among female patients and found that cigarette smoking was still the most important risk factor for laryngeal squamous cell carcinoma. With this in mind, it would be prudent to formulate policies that will reduce the prevalence and incidence of cigarette smoking among males to reduce cancer burden in this population.

Of the cases recruited, majority of them came from highland areas of Kenya's Central, Eastern, and Rift Valley provinces. This distribution is in keeping with work published by Onyango and Macharia [17] and mirrors the higher prevalence of cigarette smoking in these provinces [1, 2]. The proximity of these regions to Kenyatta National Hospital which hosts the only comprehensive public cancer treatment centre may also be a contributing factor. We can therefore postulate that the higher rates of cigarette smoking in Central and Eastern provinces, which are nearly ethnically homogenous, are responsible for the higher prevalence of laryngeal squamous cell carcinoma encountered. The contribution of genetic predisposition was not captured in this research since other regions like Coast province had high prevalence of cigarette smoking but did not have correspondingly high laryngeal cancer cases. This is an area for further research since only 62% cases of laryngeal squamous cell carcinoma have been shown to be directly linked to these two risk factors in Kenya [4].

On the other hand, there was no relation between occupation, level of education, and laryngeal cancer as opposed to findings in previous published work by Onyango and Macharia [17]. That study may not be comparable to the present one since the former had a larger sample size and included head and neck cancers in general. In other populations, low levels of education have been associated with low socioeconomic status which has been shown to confer a higher risk for laryngeal squamous cell carcinoma [18–20]. This has been attributed to higher prevalence of cigarette smoking among patients of low socioeconomic status who smoke cheaper hand rolled nonfilter cigarettes which have higher levels of carcinogens [21].

Patients who were current smokers had a significant risk for laryngeal squamous cell carcinoma in general compared to controls and there was no significant risk for laryngeal squamous cell carcinoma from environmental tobacco smoke. It should be pointed out that the respondents were few in both categories.

Earlier age of commencement of cigarette smoking has been shown at molecular level to increase risk for cancer in the aerodigestive tract [22]. This has a significant bearing in this population since it has been shown that majority of them start smoking in primary and secondary school levels [23]. Measures to reduce cigarette smoking in this age group should therefore be formulated so as to reduce cancer burden in the coming generations. On the other hand, majority of cases had smoked more than 30 pack-years increasing their risk for laryngeal cancer more than 21 times compared to controls. These facts confirm that longer duration and greater intensity of cigarette smoking portend a great risk for laryngeal SCC as shown in studies performed worldwide [24].

Alcohol consumption has been positively associated with laryngeal SCC mostly as a cofactor and also as an independent factor [10, 25]. In this study, there was an overall increased risk for laryngeal squamous cell carcinoma though the risk was significantly higher among very heavy drinkers. This finding seems to be significantly higher compared to other studies [26]. Alcohol ingestion is therefore an independent risk factor for laryngeal squamous cell carcinoma in Kenya. Such variance with other populations may be linked to the fact that majority of the alcohol consumed among the cases was traditional brew and unregulated beer.

With regard to histologic findings, majority of the patients who smoked cigarettes but did not drink alcohol developed G1 carcinoma which is not in keeping with published data [11]. The numbers involved in this study were small and therefore we may not make statistical inference based on this. Moreover, confounding factors such as GERD and human papilloma virus (HPV) may have contributed to cancer causation despite this being a controlled study as they are now acknowledged risks for head and neck squamous cell carcinoma in general including laryngeal ones. In this study, alcohol consumption predisposed to G2 carcinoma while those who were alcohol drinkers and cigarette smokers as well developed the less differentiated G3 and G4 SCC. This is in keeping with various studies done globally [11, 12, 27]. Many molecular epidemiologic studies have shown that alcohol intake and cigarette smoking cause mutation of p53 and p16 genes as well as overexpression of cyclin D1 [11, 28]. P53 gene mutation and cyclin D1 over expression among others predispose to less differentiated squamous cell carcinoma in the head and neck region [28, 29]. These may explain the histologic pattern encountered in this study. The scope of this study did not capture whether host factors influenced the histologic type and is therefore an avenue for further research.

Lastly, majority of the patients presented stage, 3 and 4 of laryngeal cancer (73.6%). From published data, tumors that are causally linked to cigarette smoking and alcohol ingestion have been shown to progress rapidly and present late [30]. These tumors are aggressive and have high recurrence rate although various other factors come into play in this region as regards late presentation as shown earlier by Oburra [31] and later by Onyango and Macharia [17]. These include their health seeking behaviour, ignorance and poverty, and lack of adequate health facilities and personnel as well as high cost of health care.

In conclusion, cigarette smoking and alcohol ingestion are important risk factors for laryngeal SCC in this population, more so in Central and Eastern provinces. Cigarette smoking without ingestion of alcohol had higher associations with well differentiated carcinoma whereas concurrent cigarette smoking and alcohol intake predisposed more to the less differentiated SCC of the larynx. The results of this study provide epidemiologic evidence that cigarette smoking and alcohol ingestion are strongly associated with poorly differentiated laryngeal squamous cell carcinoma. These tumors generally have a poorer outcome especially in the study population where cancer diagnosis, assessment, referral, management, and followup are not optimum [17].

More emphasis should be put in place to strengthen the existing tobacco control bill [32] including improved budgetary allocation for the relevant bodies to help reduce the prevalence of cigarette smoking as well as improve cancer diagnosis, treatment, and followup. A specific effort should be put to reduce cigarette smoking among school going children and college students as this will go a long way in reducing new cancer cases in future.

There were some limitations in this study. A few cases were controlled by age and sex but not geographical region of origin. This was also a hospital based study and therefore it may not reflect the true picture of the general population. Being a case-control study, recall bias may have impacted on the responses we got from the research subjects. The orthopedic trauma patients, some of whom were used in this study, may have had alcohol related accidents thus may have not been ideal controls.

## Figures and Tables

**Table 1:** Smoking history and habits.

	Controls	Cases	*P* value
Smoker			
No	94.00%	34.00%	<0.0001
Yes	6.00%	66.00%	
Cigarette type smoked			
Filtered	86.70%	69.80%	0.198
Nonfiltered	13.30%	30.20%	
Lives with smoker			
No	98.00%	96.00%	0.558
Yes	2.00%	4.00%	
Smokes in the house			
No	98.00%	94.00%	0.307
Yes	2.00%	6.00%	

**Table 2:** Tumor characteristics and stage of disease.

	Count	Column *N*%
Differentiation		
Well differentiated (G1)	19	38.00%
Moderately differentiated (G2)	17	34.00%
Poorly differentiated (G3)	10	20.00%
Undifferentiated (G4)	4	8.00%
Stage		
1	3	6.40%
2	10	20%
3	24	46%
4	13	27.60%

**Table 3:** Histological grade for concurrent smoking and alcohol intake.

Grade	Yes	No	*P* value	OR	Lower CI	Upper CI
G1	8	11				
G2	10	7	0.006	4.218	1.425	12.285
G3	8	2	<0.001	11.652	2.305	58.895
G4	3	1	0.052	7.286	0.726	73.075

## Declaration

This paper was presented during the Kenya Ear Nose and Throat Society (KENTS) Scientific Conference in May 2013 and at the 20th International Federation of Otorhinolaryngological Societies (IFOS 2013) Congress in June 2013. A summarized version was also submitted by the corresponding author to the IFOS 2013 Congress Scientific Secretariat where he won the Young Scientist of the Year Award.
